# Advanced extraction of PV parameters’ models based on electric field impacts on semiconductor conductivity using QIO algorithm

**DOI:** 10.1038/s41598-024-65091-4

**Published:** 2024-07-04

**Authors:** Ahmed S. A. Bayoumi, Ragab A. El Sehiemy, Maged El-Kemary, Amlak Abaza

**Affiliations:** 1 Mathematical and Physics Engineering Department, Faculty of Engineering, Kafrelsheikh University, Kafr ElSheikh, 33516 Egypt; 2 Akhbar ElYoum Academy, 6 October City, Egypt; 3 Electrical Engineering Department, Faculty of Engineering, Kafrelsheikh University, Kafr ElSheikh, 33516 Egypt; 4 Institute of Nanoscience & Nanotechnology, Kafrelsheikh University, Kafr ElSheikh, 33516 Egypt; 5 Nile Valley University, Fayum, Egypt

**Keywords:** Parameter extraction, Variable voltage resistances, Three PV models, Climate operation conditions, Innovative QIO optimizer, Energy science and technology, Engineering, Materials science, Mathematics and computing, Optics and photonics, Physics

## Abstract

This article presents a novel approach for parameters estimation of photovoltaic cells/modules using a recent optimization algorithm called quadratic interpolation optimization algorithm (QIOA). The proposed formula is dependent on variable voltage resistances (VVR) implementation of the series and shunt resistances. The variable resistances reduced from the effect of the electric field on the semiconductor conductivity should be included to get more accurate representation. Minimizing the mean root square error (MRSE) between the measured (I–V) dataset and the extracted (V–I) curve from the proposed electrical model is the main goal of the current optimization problem. The unknown parameters of the proposed PV models under the considered operating conditions are identified and optimally extracted using the proposed QIOA. Two distinct PV types are employed with normal and low radiation conditions. The VVR TDM is proposed for (R.T.C. France) silicon PV operating at normal radiation, and eleven unknown parameters are optimized. Additionally, twelve unknown parameters are optimized for a Q6-1380 multi-crystalline silicon (MCS) (area 7.7 cm^2^) operating under low radiation. The efficacy of the QIOA is demonstrated through comparison with four established optimizers: Grey Wolf Optimization (GWO), Particle Swarm Optimization (PSO), Salp Swarm Algorithm (SSA), and Sine Cosine Algorithm (SCA). The proposed QIO method achieves the lowest absolute current error values in both cases, highlighting its superiority and efficiency in extracting optimal parameters for both Single-Crystalline Silicon (SCS) and MCS cells under varying irradiance levels. Furthermore, simulation results emphasize the effectiveness of QIO compared to other algorithms in terms of convergence speed and robustness, making it a promising tool for accurate and efficient PV parameter estimation.

## Introduction

The reliance on finite fossil fuels like oil, coal, and natural gas creates significant challenges: they are depleting rapidly, contribute to harmful emissions, and pose health risks^1–3^. In contrast, renewable energy offers a sustainable solution: sources like sunlight, wind, and biomass constantly replenish, minimizing environmental impact and offering near-limitless potential^4,5^.

Photovoltaic (PV) panels in solar power systems directly transform sunlight into electrical energy. The energy generated can be utilized for producing electricity, stored in batteries^6–9^.

The different climate conditions in any place and during the year time even the daytime make it important to find precise PV model parameters to handle the power control issue. Efforts have been done many years before resulted in the most famous models, namely three diode model (TDM), double diode model (DDM) and single diode model (SDM)^10–13^.

Among different ways to obtain the PV models’ parameters mentioned before the metaheuristic algorithms which proved its transcendence in solving many different engineering problems. These algorithms usually prompted by variety of issues like evolution, population, physics, chemistry, human and mathematics^14–17^. Some of these issues are Marine Predators Optimizer (MPO) which is demonstrated and used alongside FOPID for enhancing AVR controller^18^, a Fractional-Order PID Controller with an Improved MPO for an automatic voltage regulator system^19^, bald eagle search optimizer was used for determining optimal parameters of a solid oxide fuel cell model^20^, solar cell modelling with high accuracy using genetic neural network-based algorithms^21^, image edge identification Using the Ant Colony Algorithm of Fractional Order^22^, real-time optimization of a fractional order controller for industrial robots using digital twins^23^ and optimal DG positioning in a radial distribution network using an improved real coded genetic algorithm.

Table 1 summaries the recent application of parameter estimation problems and the associated solution methodologies. The drawbacks of meta-heuristic methods, even with the existence of many merits, like the high dimension of the target PV modeling process may leads to local optimal solution, due to many climate changes, the modules may be degraded and therefore the parameters coordination to be difficult to predicted.

**Table 1:** A survey of the optimization algorithms that have been used in the five 2 years.

Ref.	Used algorithm	Model			Fitness function
		SDM	DDM	TDM	
^24^	Improved gradient-based optimizer	✓	✓	–	RMSE
^25^	Equilibrium, coot bird and artificial ecosystem optimizations				
^26^	Artificial ecosystem-based optimization			✓	RMSE
^27^	Simplified swarm				
^28^	Hybrid African vultures–grey wolf optimizer			✓	ASE, QCE
^29^	Modified social network search algorithm combined with the Secant method	✓			RMSE
^30^	Improved stochastic fractal search	✓			RMSE
^31^	Random learning gradient- optimizer	✓	✓	✓	
^32^	Comprehensive learning Rao-1	✓	✓		
^33–35^	Differential evolution algorithm	✓	✓		
^36^	Arithmetic optimizer	✓	✓	✓	
^37^	Fractional chaotic ensemble particle swarm optimizer				
^38^	Supply demand algorithm			✓	
^39^	Spotted hyena optimizer	✓	✓	✓	
^40^	Northern Goshawk Optimizer			✓	

This study leverages a novel mathematical algorithm called Quadratic Interpolation Optimization (QIO)^14^. QIO is used to obtain accurate parameters for a novel PV models named as variable voltage resistor (VVR) applied to (R.T.C. France) silicon under normal operating conditions and on Q6-1380 of area 7.7 cm^2^ of multi-crystalline silicon MCS operating under low radiation conditions demonstrating the effect of grain boundaries^41^. The salient issues of this study are summarized as:

i. The use of QIQ for the parameter extraction of PV modules at different climate changes.
ii. Comparison with several optimizers is carried out and well as with the traditional TDM with TDM-VVR.
iii. Corroboration analysis for the tested PV modules by experimentation.

The following parts of this study are planned as follows: In “Basics of VVR dependent PV model” section, the basics of VVR dependent model is presented. In “Quadratic Interpolation Optimization (QIO)” section, the structure of the suggested optimization methods is constructed. The numerical simulations are presented in “Results” section. The last section concludes the paper findings.

## Basics of VVR dependent PV model

Varying operation conditions have highlighted the need for more advanced models, considering the diverse employed in the PV cells fabrication. A lot of models have been used to illustrate the inherent features of PV cells. As mentioned before the TDM is the most accurate model among the SDM, DDM, and TDM. The effect of the electric field on the semiconductor conductivity should be included to get more accurate representation. In this work, the VVR PV model is presented as follows:

As the cell illuminated, charge generation occurs, leading to a current denoted as I_PV_ running in parallel to the diode representing the PN junction^42^. In practical terms, an additional diode is introduced in parallel to the first diode to address space charge recombination^43^. The implications of recombination and large leakage in the defect region are considered by the diode parallel to the past two diodes^44^.

**Figure 1:**
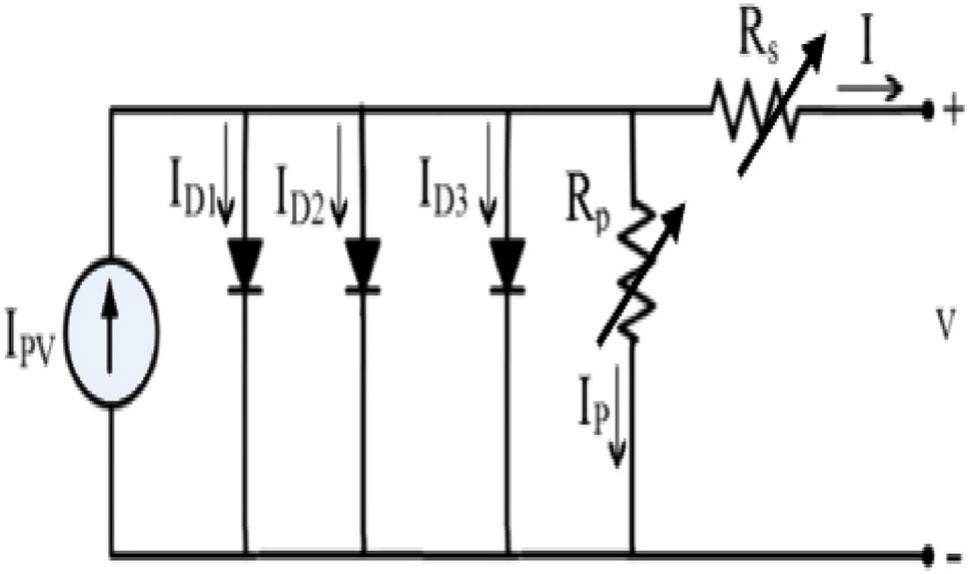
VVR TDM for SSC.

The resistance (R_P_) which considers the current leakage of PN junctions is composed of the partial current in the short circuit path near the cell's borders associated to the semi-conductor several layers and non-idealities, in addition to a resistance in series (R_s_) that considers the implications of electrodes surfaces contact, electrodes resistance, flowing current resistance and silicon. Figure 1 shows the proposed model.

To account the electric field implications the two mentioned resistances can be expressed as follow^28,29^:

1 $$R_{s} = R_{so} (1 + K_{s} \cdot V),$$

2 $$R_{p} = R_{po} (1 + K_{p} \cdot V),$$

where R_os_, R_po_ are the series and parallel resistance at zero output voltage and K_s_, K_p_ are real constants. The overall equation is:

3 $$\begin{aligned} I = & I_{ph} - I_{s1} \left[ {\exp \left( {\frac{{q\left( {V_{{}} + R_{so} (1 + K_{s} \cdot V) \cdot I} \right)}}{{(m_{1} ) \cdot k_{B} \cdot T}}} \right) - 1} \right] - I_{s2} \left[ {\exp \left( {\frac{{q\left( {V_{{}} + R_{so} (1 + K_{s} \cdot V) \cdot I_{s} } \right)}}{{(m_{2} ) \cdot k_{B} \cdot T}}} \right) - 1} \right] \\ & - I_{s3} \left[ {\exp \left( {\frac{{q\left( {V_{{}} + R_{so} (1 + K_{s} \cdot V) \cdot I} \right)}}{{(m_{3} ) \cdot k_{B} \cdot T}}} \right) - 1} \right] - \frac{{V_{{}} + R_{so} (1 + K_{s} \cdot V) \cdot I}}{{R_{po} (1 + K_{p} \cdot V)}}, \\ \end{aligned}$$

where I_s1_, I_s2_ and I_s3_ represent reverse saturation currents of the three diodes respectively, m_1_, m_2_ and m_3_ represent ideality factors of the three diodes respectively, V represents the output voltage, the temperature in Kelvin denoted by T and Boltzmann’s constant is denoted by K_B_.

**Figure 2:**
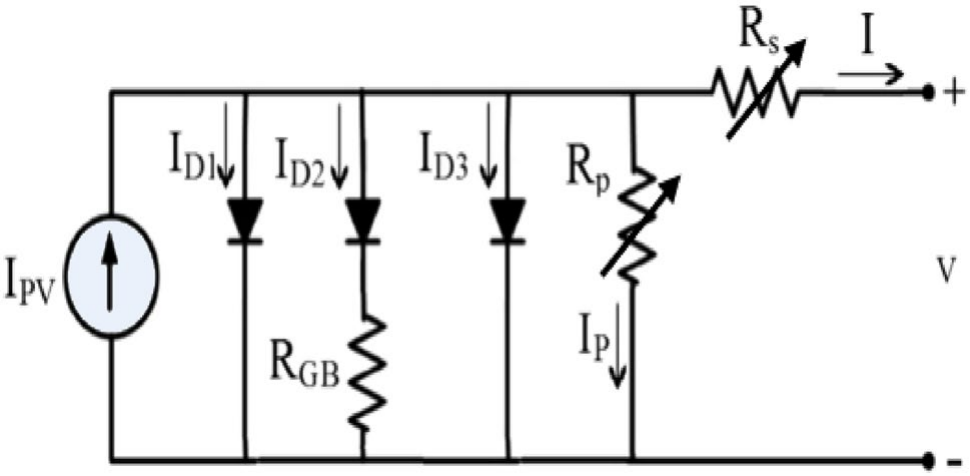
VVR TDM for MCSSC.

The significance of multi-crystalline silicon solar cell (MCSSC) necessitates the development of more complex models that account for the different implications of grains and grain boundaries, in addition to leakage, recombination, and other implications^45,46^. A series resistance R_GB_ is associated to the second diode to account for resistivity near grain boundaries, which is greater than resistance within crystallites which appears especially at low radiation^47^. The overall model is shown in Fig. 2 and the current equation is represented as:

4 $$\begin{aligned} I = & I_{ph} - I_{s1} \left[ {\exp \left( {\frac{{q\left( {V + R_{so} (1 + K_{s} \cdot V) \cdot I} \right)}}{{(m_{1} ) \cdot k_{B} \cdot T}}} \right) - 1} \right] - I_{s2} \left[ {\exp \left( {\frac{{q\left( {V_{{}} + R_{so} (1 + K_{s} \cdot V) \cdot I - I_{D2 } R_{GB} } \right)}}{{(m_{2} ) \cdot k_{B} \cdot T}}} \right) - 1} \right] \\ & - I_{s3} \left[ {\exp \left( {\frac{{q\left( {V + R_{so} (1 + K_{s} \cdot V) \cdot I} \right)}}{{(m_{3} ) \cdot k_{B} \cdot T}}} \right) - 1} \right] - \frac{{V + R_{so} (1 + K_{s} \cdot V) \cdot I}}{{R_{po} (1 + K_{p} \cdot V)}}. \\ \end{aligned}$$

## Quadratic Interpolation Optimization (QIO)

An innovative meta-heuristic algorithm prompted by mathematics, quadratic interpolation optimization algorithm (QIOA) is used to solve engineering and numerical optimization problems. In order to find the minimizer of the quadratic function formed by any three points more effectively, this method gets around the drawbacks of the conventional quadratic interpolation method. The generalized quadratic interpolation (GQI) technique is an exciting searching mechanism that the QIOA employs to address a variety of problems. The minimizer supplied by the GQI method enables the QIOA to explore an exciting area in unexplored areas and exploit the best solutions in promising regions. This searching mechanism provides strategies for both exploration and exploitation^14^.

### Exploration strategy

The suggested QIOA’s exploration tactics is determined by the GQI method. The GQI method is used in conjunction with the current individual and two randomly selected individuals from the current population to find promising areas and to obtain the minimizer of the interpolation function formed by three positions. In the meantime, the new candidate solution is created using the third person who was randomly selected from the current population. The current individual’s position is modified as:

5 $$p_{i} (z + 1) = x_{i,rand1,rand2}^{*} (z) + w_{1} \cdot (x_{rand3} (z) - x_{i,rand1,rand2}^{*} (z)) + round(0.5 \cdot (0.05 + r_{1} )) \cdot \log \frac{{r_{2} }}{{r_{3} }},$$

6 $$x_{i,rand1,rand2}^{*} (z) = GQI(x_{i} (z),x_{rand1} (z),x_{rand2} (z),fit(x_{i} ),fit(x_{rand1} (z)),fit(x_{rand2} (z))),$$

where x_rand1_, x_rand2_ and x_rand3_ are the current population of different individuals randomly chosen positions, r_1_, r_2_ and r_3_ are random values in range of (0,1), and fit (·) displays the magnitude of the function fitness. The GQI function that computes the minimizer of interpolation function is GQI (x_i_(z), x_rand1_(z), x_rand2_(z), fit(x_i_(z)), fit(x_rand1_(z)), fit(x_rand2_(z))) which is formed by (x_i_, fit(xi)), (x_rand2_, fit(x_rand2_)), and (x_rand3_, fit(x_rand3_). The search range can effectively be broadened by r_1_, r_2_ and r_3_ and a promising area can be placed with the help of GQI.

A significant variable known as the exploration weight w_1_ is added in order to make the algorithm able to initially be carrying out exploration, subsequently balancing exploration, and exploitation, and then converging to optimal solution. An adaptable factor b may be employed to modify w_1_. They can be described as follows:

7 $$w_{1} = 3n_{1} \left( {0.7 + 0.15\left( {\cos \left( {\frac{5\pi z}{Z}} \right) + 1} \right)} \right)\cos \left( {\frac{\pi z}{{2Z}}} \right),$$

where n_1_ is a standard normal distribution governed parameter, z represents iteration’s order, and Z represents the iterations upper limit. This combination supports avoidance of early convergence and improves the algorithm’s search capability.

### Exploitation strategy

During the exploitation phase in QIO, the GQI method is used with two randomly selected individuals from present population and the best individual found in order to generate an improved solution. The algorithm’s ability to identify the optimum global solution in the area surrounding the minimizer is encouraging. As a result, when QIO executes exploitation, the present individual’s place is modified as follows:

8 $$p_{i} (z + 1) = x_{bestrand1,rand2}^{*} (z) + w_{1} \cdot \left( {x_{best} (z) - round(1 + rand) \cdot \frac{Ub - Lb}{{Ub^{rD} - Lb^{rD} }} \cdot x_{i}^{rD} (z)} \right),$$

9 $$x_{best,rand1,rand2}^{*} (z) = GQI\left( {x_{best} (z),x_{rand1} (z),x_{rand2} (z),fit(x_{best} ),fit(x_{rand1} (z)),fit(x_{rand2} (z))} \right),$$

10 $$w_{2} = 3n_{2} \left( {1 - \frac{z - 1}{Z}} \right),$$

where n2 is a standard normal distribution governed parameter and rD represents an integer randomly selected in range of [1, d], Ub^rD^ and Lb^rD^ are upper and lower boundaries at the (rD)^th^ dimension, and the exploitation weight represented by the adaptive coefficient w_2_. It is easily seen that values of w_2_ decrease over time as number of iterations rises, which significantly improves convergence precision.

Equation (4) is applied to modify the ith individual by exploring the neighborhood of x*_best, rand1, rand2_ with respect to x_best_ based on the random perturbation from $x_{i}^{rD}$. In Eq. (9) the GQI method, the best individual obtained thus far, x_best_, and two selected at random individuals, x_rand1_ and x_rand2_, are used to produce the minimizer x*_best, rand1, rand2_, which is usually superior to the three individuals. This approach facilitates successful exploitation without encountering stagnation in the algorithm. Following the completion of the exploitation and exploration strategies, the location of the ith individual is modified using Eq. (11):

11 $$\begin{gathered} \hfill \\ x_{i} (z + 1) = \left\{ {\begin{array}{*{20}l} {x_{i} (z)} \hfill & {fit(x_{i} (z)) \le fit(p_{i} (z + 1))} \hfill \\ {p_{i} (z + 1)} \hfill & {fit(x_{i} (z)) > fit(p_{i} (z + 1))} \hfill \\ \end{array} } \right.. \hfill \\ \end{gathered}$$

The ith individual’s location is substituted with its candidate location if fitness value of it is greater than that of the current one, otherwise, nothing will change.

A set of random solutions are generated to start the QIOA. When rand > 0.5, the algorithm gets the minimizer of GQI function using two randomly chosen individuals with current individual by Eq. (6) and explores using Eq. (5), if rand ≤ 0.5, the algorithm uses Eq. (9) to find the minimizer of GQI function with the best individual obtained so far and two selected at random individuals, and Eq. (8) for exploitation. When exploitation or exploration is completed, Eq. (11) is used to modify the best solution obtained thus far.

**Figure 3:**
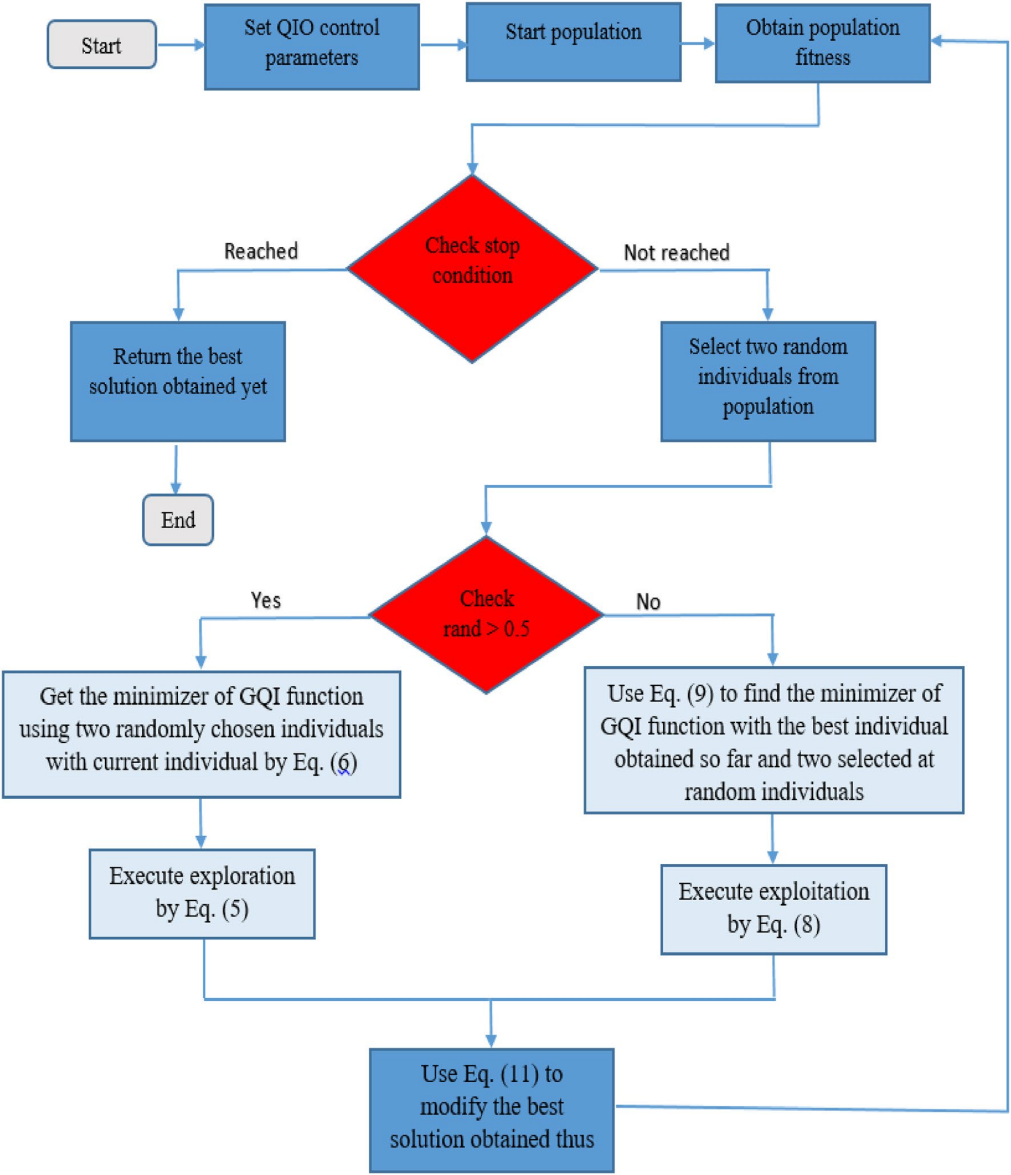
QIOA flowchart.

Finally, the QIOA terminates when a pre-defined termination criterion is satisfied, and the best solution obtained thus far is provided. The primary objective of the current optimization problem is to minimize the mean root square error (MRSE) between the measured (I–V) dataset and the extracted (I–V) curve derived from the proposed electrical model. The unknown parameters of the proposed PV models under the considered operating conditions are identified and optimally extracted using the proposed QIOA. Two types of PV are considered, one operating under normal radiation conditions and the other under low radiation conditions. VVR TDM is proposed for (R.T.C. France) silicon PV operating at normal radiation, and eleven unknown parameters are optimized (${\text{I}}_{{{\text{PV}}}} ,\, {\text{I}}_{{{\text{s1}}}} ,\,{\text{I}}_{{{\text{s2}}}} ,\,{\text{I}}_{{{\text{s3}}}} ,\,{\text{m}}_{{1}} ,\,{\text{m}}_{{2}} ,\,{\text{m}}_{{3}} ,\,{\text{R}}_{{{\text{Po}}}} ,\,{\text{R}}_{{{\text{so}}}} { },\,K_{p} {\text{ and }}K_{s} {)}$. In addition, twelve unknown parameters are optimized for Q6-1380 of area 7.7 cm2 of multi-crystalline silicon solar cell MCSSC operating at low radiation (${\text{I}}_{{{\text{PV}}}} , {\text{I}}_{{{\text{s1}}}} , {\text{I}}_{{{\text{s2}}}} ,{\text{I}}_{{{\text{s3}}}} , {\text{m}}_{1} ,{\text{m}}_{2} ,{\text{m}}_{3} , {\text{R}}_{{{\text{Po}}}} , {\text{R}}_{{{\text{so}}}} , K_{p} , K_{s} {\text{ and }}R_{GB} ).$ The QIOA is employed to extract accurate model parameters with minimum execution time. Figure 3 depicts flowchart of the proposed QIOA.

The objective function of the optimizing problem is defined with its constraints as follows:

12 $$MRSE=\sqrt{\frac{1}{N}\sum_{k=1}^{N}{\left[I\left(k\right)-{I}_{e}\left(k\right)\right]}^{2}},$$

13 $$OF=\text{min}\left(MRSE\right),$$

$$subject \,to:$$

14 $${\text{I}}_{\text{pv}}^{\text{min}}{\le \text{I}}_{\text{pv}}\le {\text{I}}_{\text{pv}}^{\text{max}}$$

15 $${\text{I}}_{\text{si}}^{\text{min}}{\le \text{I}}_{\text{si}}\le {\text{I}}_{\text{si}}^{\text{max}}\text{ for i}=\text{1,2}, \, {\text{and}} \, 3,$$

16 $${\text{m}}_{\text{i}}^{\text{min}}{\le \text{m}}_{\text{i}}\le {\text{m}}_{\text{i}}^{\text{max}}\text{ for i}=\text{1,2}, \, {\text{and}} \, 3,$$

17 $${\text{R}}_{\text{Po}}^{\text{min}}{\le \text{R}}_{\text{Po}}\le {\text{R}}_{\text{Po}}^{\text{max}},$$

18 $${\text{R}}_{\text{so}}^{\text{min}}{\le \text{R}}_{\text{so}}\le {\text{R}}_{\text{so}}^{\text{max}},$$

19 $${\text{K}}_{\text{p}}^{\text{min}}{\le \text{K}}_{\text{p}}\le {\text{K}}_{\text{p}}^{\text{max}},$$

20 $${\text{K}}_{\text{s}}^{\text{min}}{\le \text{K}}_{\text{s}}\le {\text{K}}_{\text{s}}^{\text{max}},$$

21 $${\text{R}}_{\text{GB}}^{\text{min}}{\le \text{R}}_{\text{GB}}\le {\text{R}}_{\text{GB}}^{\text{max}},$$

where *I*_*e*_ represents the experimental current with N samples.

## Results

The performance of the proposed QIOA is evaluated by extracting the optimal parameters of two case studies of a new VVR TDM of PV cells: The proposed QIO is used to extract the optimal model parameters of the two PV types. For the case, Table 2 reports the intervals boundaries provided in the literature for RTC France solar cell^38,49^. In Tables 3 and 4, the boundaries are considered according to references^41,50^.

**Table 2:** Optimal parameters of Case 1with different PV models using QIOA.

Parameter	Limits		Case 1	
	Lower boundary	Upper boundary	TDM	VVR-TDM
Ipv (A)	0	1	0.762	0.762
Is1 (μA)	0	1	3.76E−08	1.13E−06
Is2 (μA)	0	1	1.39E−06	9.50E−07
Is3 (μA)	0	1	8.60E−07	5.13E−07
m1	1	2	1.832	1.967
m2	1	2	1.656	1.764
m3	1	2	1.930	1.580
RPo	0	100	71.831	87.424
Rso	0	0.5	0.027	0.028
Ks	− 0.01	0.5	–	− 3.54E−04
Kp	− 0.01	1.5	–	− 0.014
RGB	0	2	–	1.07
RMSE			4.69E−03	3.71E−03

**Table 3:** Comparison of extracted parameters of Case 1 with different PV models using QIOA.

Parameters	Boundary		TDM	VVR-TDM
	Lower	Upper		
Ipv (A)	0.7608	0.7609	0.7608	0.76081
Is1 (A)	1.00E−08	1.00E−07	9.65E−08	9.79E−08
Is2 (A)	1.00E−13	1.00E−11	7.95E−12	3.59E−12
Is3 (A)	1.00E−05	0.0001	1.76E−05	3.36E−05
m1	1	2	1.373	1.373533
m2	1	4	1.449	2.006322
m3	1	5.5	2.737	3.01504
RPo	60	75	64.731	73.78624
Rso	0.0035	0.04	0.039	0.03927
Ks	− 0.001	0.0001	–	− 0.00019
Kp	− 0.2	0.25	–	− 0.03316
RMSE			7.60E−04	7.537E−04

**Table 4:** Comparison of optimal parameters of Case 2 with different PV models by QIOA.

Parameters	Boundary		TDM	VVR-TDM
	Lower	Upper		
Ipv (A)	0	2.10E−02	1.94E−02	1.94E−02
Is1 (A)	1.00E−12	1.00E−03	1.11E−08	1.95E−07
Is2 (A)	1.00E−09	1.00E−02	2.36E−05	2.39E−04
Is3 (A)	1.00E−12	1.00E−02	7.13E−05	1.01E−04
m1	1	2	1.858	1.983
m2	2	3.5	2.877	2.152
m3	3.5	5	4.044	3.518
RPo	600	1500	793.532	1293.320
Rso	0.04	2	0.870	1.003
Ks	− 0.01	0.5	–	0.289
Kp	− 0.01	1.5	–	0.642
RGB	0	2	–	1.072
RMSE			1.44E−05	1.386E−05

### Case study 1

In this case, R.T.C. France silicon solar cell with 57 mm diameter commercial is tested under normal operating conditions (1000 W/m^2^ at 33 °C). The experimental current–voltage dataset is taken from literature^48^.

### Case study 2

This case involves an MCSSC Q6-1380 with an area of 7.7 cm^2^ to demonstrate the accuracy of the proposed VVR TDM under room temperature (27 °C) and low irradiation level (98.4 W/m^2^). The experimental current–voltage dataset is taken from literature^51^.

The objective function in this study is the Root Mean Square Error (RMSE) between the experimental (I-V) dataset and the estimated current of the PVs. The efficacy of the QIOA is tested and compared to four well-known optimizers (GWO, PSO, SSA, and SCA). The population size and number of iterations for all competitive optimizers are taken the same for a fair comparison.

The parameters of the VVR TDM of both case studies are considered as the control variables of the optimized problem. The population size and the maximum number of iteration are set at 150 agents and 400 iterations, respectively. All reported results in this study are carried out using the MATLAB R2016b software on a PC with Intel(R) Core (TM)i3-CPU M370@2.4 GHz 3 GB (RAM). The value objective function (RMSE) over 100 runs of the algorithms can be used to prove the robustness of the proposed QIOA compared to the four chosen competitive algorithm.

In Table 2, the optimal parameters are extracted based on the QIQA for both models under the given boundaries. This table reports the optimal extracted parameters for Case 1 in both the TDM and VVR TDM, estimated using the QIOA according to the lower and upper limits of the PV parameters in Refs.^38,49^. The simulation results in this table explains that the extracted parameters agree with experimental with lower RMSE value when the proposed VVR TDM is used. In Case 1, the RMSE is 3.71E-03 with VVR TDM compared to 4.69E-03 with TDM.

Tables 3 and 4 report the optimal extracted parameters for Case 1 and Case 2 in both the TDM and VVR TDM, estimated using the QIOA according to the upgraded lower and upper limits of the PV parameters^41,50^. The simulation results in this table explains that the extracted parameters agree with experimental with lower RMSE value when the proposed VVR TDM is used. In Case 1, the RMSE is 7.537E−04 with VVR TDM compared to 7.60E−04 with TDM. Moreover, the RMSE is 1.386E−05 with VVR TDM compared to 1.44E−05 with TDM, in Case 2.

Tables 5 and 6 provide an evaluation of the accuracy of QIOA in comparison to GWO, PSO, SSA and SCA optimizers. The optimal parameters along with the corresponding RMSE values obtained via each optimizer are reported. The rank of RMSE shows that QIOA has the least value (7.538E−04) following PSO (7.561E−04), GWO (7.568E−04), SSA (8.162E−04), and SCA (9.271E−04) for Case 1. For Case 2, again QIOA has the least value (1.386E−5) following PSO (1.491E−5), GWO (1.531E−5), SCA (1.709E−5), and SSA (1.724E−5).

**Table 5:** Optimal PV estimated parameters of of VVR TDM for SSC using QIOA compared with competitive optimizers (Case study 1).

Method	I_PV_ (A)	I_S1_ (A)	I_S2_ (A)	I_S3_ (A)	m_1_	m_2_	m_3_	R_Po_ (Ω)	R_So_ (Ω)	K_S_	K_p_ (A)	RMSE	Rank
QIOA	0.7608	9.79E−08	3.59E−12	3.36E−05	1.374	2.006	3.015	73.78	0.039	− 1.88E−04	− 3.32E−02	7.538E−04	1
GWO	0.7608	9.75E−08	1.69E−12	2.94E−05	1.373	1.503	2.959	72.53	0.039	− 2.18E−05	− 3.60E−02	7.568E−04	3
PSO	0.7609	1.00E−07	1.00E−11	5.24E−05	1.375	4.000	3.283	75.00	0.039	− 1.00E−03	− 4.46E−02	7.561E−04	2
SSA	0.7608	6.52E−08	5.77E−13	1.39E−05	1.344	1.398	2.486	71.79	0.039	− 5.93E−06	1.55E−01	8.162E−04	4
SCA	0.7609	8.46E−08	5.31E−12	9.82E−05	1.360	2.941	3.698	73.34	0.040	− 5.19E−04	− 1.95E−01	9.271E−04	5

**Table 6:** Optimal PV estimated parameters of VVR TDM for MCSSC using QIOA compared with competitive optimizers (Case study 2).

Method	I_PV_ (A)	I_S1_ (A)	I_S2_ (A)	I_S3_ (A)	m_1_	m_2_	m_3_	R_Po_ (Ω)	R_So_ (Ω)	K_S_	K_p_ (A)	R_GB_	RMSE	Rank
QIOA	1.94E−2	1.95E−7	2.39E−4	1.01E−4	1.98	2.15	3.52	1293.32	1.00	0.289	0.642	1.072	1.386E−5	1
GWO	1.94E−2	6.88E−12	2.79E−4	1.11E−4	1.92	2.32	3.50	1203.49	0.58	− 0.006	0.298	1.723	1.531E−5	3
PSO	1.94E−2	1.0E−12	1.85E−4	1.13E−4	2.00	3.50	3.50	1000.00	0.07	0.500	1.500	0.000	1.491E−5	2
SSA	1.94E−2	2.11E−9	4.09E−5	1.16E−4	1.40	2.48	3.53	1021.27	0.21	0.211	0.962	1.893	1.724E−5	5
SCA	1.93E−2	1.33E−12	3.93E−9	1.13E−4	1.21	2.00	3.50	1236.33	0.06	− 0.007	0.013	0.000	1.709E−5	4

**Figure 4:**
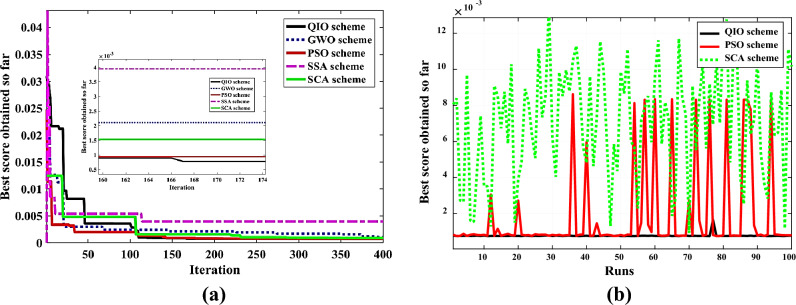
Convergence rates and robustness of VVR TDM for SSC using QIOA compared with competitive optimizers (Case study 1): (**a**) Convergence rate; (**b**) Robustness.

**Figure 5:**
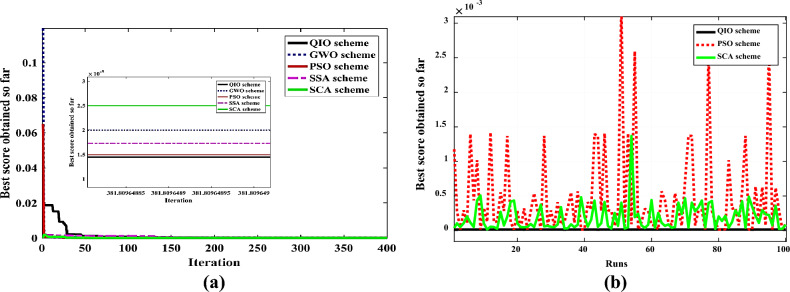
Convergence rates and robustness of VVR TDM for MCSSC using QIOA compared with competitive optimizers (Case study 2): (**a**) Convergence rate; (**b**) Robustness.

**Figure 6:**
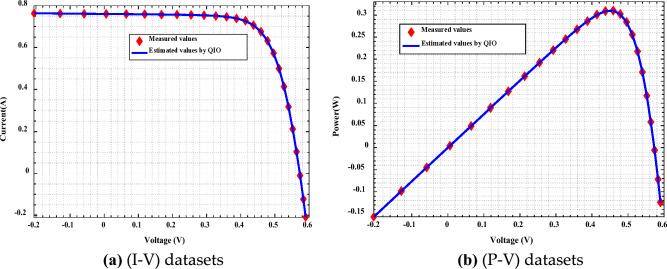
Measured and estimated data of VVR TDM for SSC using QIOA (Case study 1).

**Figure 7:**
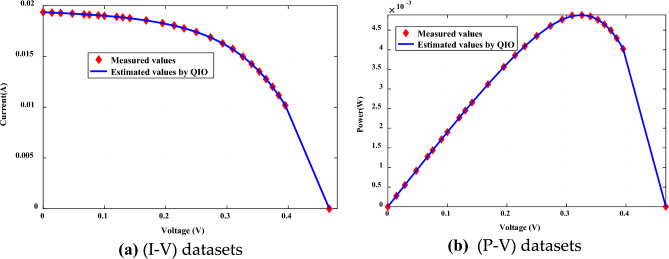
Measured and estimated data of VVR TDM for MCSSC using QIOA (Case study 2).

The convergence rate and robustness of QIOA compared to other optimizers are explained in Figs. 4 and 5 for Cases 1 and 2, respectively. The results emphasize the effectiveness of QIO compared to others. The extracted optimal parameters are used to obtain (I–V) and (P–V) curves of PVs. Figures 6 and 7 illustrate the constructed estimated curves for both case studies with the proposed VVR TDM and using QIOA. It is cleared that I–V and P–V curves for Case 1 (SSC at normal irradiance) and Case 2 (MCSSC at low irradiance) are highly closest to the measured dataset, which emphasizes the efficiency of both the suggested model and the optimization method in modeling the PV performance. To further investigate the accuracy and effectiveness of QIOA, statistical analysis with minimum, maximum, mean, standard deviation (STD), variance and median of RMSE, is implemented over 100 runs for the two studied cases VVR TDM of PV. The statistical indices prove the QIOA accuracy.

Tables 7 and 8 report the statistical indicies of Cases 1 and 2, respectively. It is cleared that the proposed QIO-based extracted parameters method has the minimum RMSE over all optimizers. PSO gives the second minimum RMSE followed by GWO optimizer. The STD indicates that QIOA can realize higher reliability in optimized problem, as noticed from Tables 7 and 8 where QIOA gives the best STD, 8.913E−05 and 1.869E−07, for Case 1 and Case 2 respectively. The PSO achieves the second best RMSE in Cases 1 and 2, while GWO realizes the second best STD in Case 1 and SSA achieves the second best STD in Case 2. All simulation results indicate that the proposed QIOA outperforms the other four competitive optimizers. It is accurate, effective, and reliable in obtaining optimal parameters of TDM and VVR TDM PVs at different operating conditions.

**Table 7:** Statistical indices of VVR TDM for SSC using QIOA compared with competitive optimizers (Case study 1).

Metric	QIO	GWO	POS	SSA	SCA
Min.	7.538E−04	7.568E−04	7.561E−04	8.162E−04	9.271E−04
Max.	1.654E−03	8.585E−03	8.621E−03	9.145E−03	1.357E−02
Mean	7.755E−04	3.249E−03	1.852E−03	3.611E−03	7.114E−03
STD	8.913E−05	3.189E−03	2.470E−03	1.704E−03	3.086E−03
Variance	7.945E−09	1.017E−05	6.101E−06	2.905E−06	9.525E−06
Median	7.638E−04	9.368E−04	8.149E−04	3.199E−03	7.353E−03

**Table 8:** Statistical indices of VVR TDM for MCSSC using QIOA compared with competitive optimizers (Case study 2).

Metric	QIO	GWO	POS	SSA	SCA
Min.	1.386E−05	1.531E−05	1.491E−05	1.724E−05	1.709E−05
Max.	1.483E−05	1.351E−03	3.264E−03	4.537E−04	1.369E−03
Mean	1.470E−05	5.887E−05	4.775E−04	1.503E−04	1.879E−04
STD	1.869E−07	1.355E−04	6.384E−04	9.542E−05	1.903E−04
Variance	3.494E−14	1.835E−08	4.076E−07	9.104E−09	3.623E−08
Median	1.475E−05	3.161E−05	2.847E−04	1.344E−04	1.055E−04

**Figure 8:**
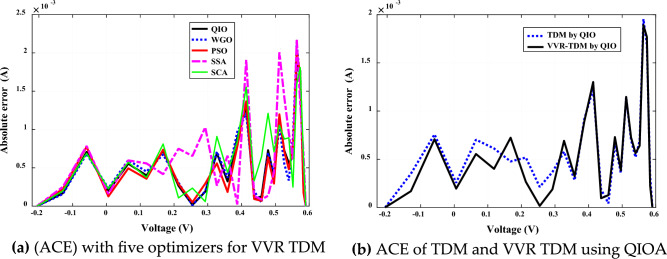
Absolute current error (ACE) for SSC (Case study 1).

**Figure 9:**
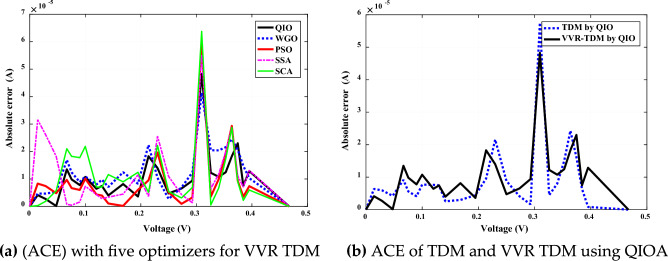
Absolute current error (ACE) for SSC (Case study 2).

In-depth investigation of the absolute current error (ACE) using QIO and four competitive optimizers for VVR TDM is executed for Case 1 and Case 2. Figures 8a and 9a illustrate this error for both case studies. It is noticed that the proposed QIO optimizer in both Cases has the lowest absolute current error values. Figures 8b and 9b illustrate that ACE error is lower for VVR TDM using QIO optimizer. It is noticed that the proposed QIO optimizer in both Cases has the lowest absolute current error values. This also proves the superiority, and efficiency of the proposed QIO optimizer in extracting the optimal parameters of both SSC and MCSSC at normal and low irradiance, respectively.

## Conclusions

This paper has introduced a novel formulation of the parameter extraction that considers the impact of the electric field on the semiconductor conductivity that is emulated by the variable voltage resistances for shunt and series resistances of photovoltaic models. The quadratic interpolation optimization algorithm has been developed to solve the modified formulation. The main objective function is aimed at minimizing the mean root square error between the measured dataset and the extracted curve. The unknown parameters of the proposed PV models were extracted under different climate conditions. Two of PV types are employed with normal and low radiation conditions for the R.T.C. France silicon solar cell (SSC) and for Q6-1380 of area 7.7 cm^2^ of multi-crystalline silicon solar cell (MCSSC) operating at low radiation, respectively. The effectiveness of QIOA was tested and compared to GWO, PSO, SSA, and SCA. The proposed QIOA has the lowest absolute current error values in both cases studied. All simulation results indicate that the proposed QIO outperforms the other four competitive optimizers. It is accurate, effective, and reliable in obtaining optimal parameters of TDM and VVR TDM PVs at different operating conditions. Additionally, the superiority, and efficiency of the proposed QIO optimizer in extracting the optimal parameters of both SSC and MCSSC at normal and low irradiance, respectively are proven. Moreover, the simulation results emphasize the effectiveness of QIO compared to others in terms of the convergence and robustness degrees.

## Data Availability

All data generated or analyzed during this study are included in this published article [and its Supplementary Information files.
